# Label-Free Visualization and Tracking of Gold Nanoparticles in Vasculature Using Multiphoton Luminescence

**DOI:** 10.3390/nano10112239

**Published:** 2020-11-12

**Authors:** Sean Burkitt, Mana Mehraein, Ramunas K. Stanciauskas, Jos Campbell, Scott Fraser, Cristina Zavaleta

**Affiliations:** 1 Department of Biomedical Engineering, University of Southern California, 1042 Downey Way, Los Angeles, CA 90089, USA; sburkitt@usc.edu (S.B.); mehraein@usc.edu (M.M.); joscampb@usc.edu (J.C.); sfraser@provost.usc.edu (S.F.); 2 Michelson Center for Convergent Biosciences, 1002 Child’s Way, Los Angeles, CA 90089, USA; 3 Bridge Institute, University of Southern California, 1002 Child’s Way, Los Angeles, CA 90089, USA; 4 Nikon Instruments Inc., 1300 Walt Whitman Road, Melville, NY 11747-3064, USA; ramunas.stanciauskas@nikon.com; 5 Department of Biological Sciences, University of Southern California, 3616 Trousdale Parkway, Los Angeles, CA 90089, USA

**Keywords:** gold nanoparticles, multiphoton microscopy, label-free imaging, multiphoton luminescence, real time imaging

## Abstract

Gold nanoparticles continue to generate interest for use in several biomedical applications. Recently, researchers have been focusing on exploiting their dual diagnostic/therapeutic theranostic capabilities. Before clinical translation can occur, regulatory agencies will require a greater understanding of their biodistribution and safety profiles post administration. Previously, the real-time identification and tracking of gold nanoparticles in free-flowing vasculature had not been possible without extrinsic labels such as fluorophores. Here, we present a label-free imaging approach to examine gold nanoparticle (AuNP) activity within the vasculature by utilizing multiphoton intravital microscopy. This method employs a commercially available multiphoton microscopy system to visualize the intrinsic luminescent signal produced by a multiphoton absorption-induced luminescence effect observed in single gold nanoparticles at frame rates necessary for capturing real-time blood flow. This is the first demonstration of visualizing unlabeled gold nanoparticles in an unperturbed vascular environment with frame rates fast enough to achieve particle tracking. Nanoparticle blood concentration curves were also evaluated by the tracking of gold nanoparticle flow in vasculature and verified against known pre-injection concentrations. Half-lives of these gold nanoparticle injections ranged between 67 and 140 s. This label-free imaging approach could provide important structural and functional information in real time to aid in the development and effective analysis of new metallic nanoparticles for various clinical applications in an unperturbed environment, while providing further insight into their complex uptake and clearance pathways.

## 1. Introduction

Nanoparticles have great potential for several biomedical applications and are currently being used as drug delivery vehicles, treatment response monitors, and in early-stage disease diagnostics [1,2,3,4]. As imaging contrast agents, nanoparticles have distinct advantages over their small molecule counterparts and are exploited in a wide variety of imaging modalities [5,6]. In particular, gold nanoparticles have been used as multimodal imaging agents with applications in computed tomography (CT), photoacoustic and Raman imaging [6,7,8,9]. Gold nanoparticles are also increasingly utilized as “theranostic” agents, combining therapeutic uses such as thermal ablation with other diagnostic imaging capabilities [10,11]. In clinical trials, gold has been used for the treatment of inoperable tumors using gold nanoparticles conjugated with tumor necrosis factor [12]. Other ongoing clinical trials include the thermal ablation of head and neck tumors, lung nodules, and prostate cancer using gold–silica nanoparticles [13,14,15,16]. They have also been recently FDA-approved for the treatment of acne [17]. Gold nanoparticles are at an inflection point for clinical translation; however, this transition will require a more thorough assessment of their in vivo distribution and fate.

Intravital microscopy (IVM) has emerged as a profound imaging modality for gaining insight into dynamic processes on the cellular and subcellular level, particularly for visualizing nanoparticles [14,18,19,20,21]. A primary challenge in the clinical translation of nanoparticles is identifying and understanding their biological fate, which relies on the visualization of these processes. For gold nanoparticles, characterizing their long-term systemic effects is essential to gain clinical acceptance. Identifying and tracking gold nanoparticles within the vascular environment could provide new insights to enable their clinical translation. IVM has previously enabled the observation of several fundamental nanoparticle processes such as extravasation and tumor accumulation in real time within a living organism [18]. With the increased interest in utilizing metallic-based nanoparticles for their dual diagnostic/therapeutic capabilities, a greater understanding is necessary with regard to their biodistribution and safety profiles post administration. Most nanoparticles used in healthcare today are administered intravenously (IV); however, little is known about the biological interactions that take place with these nanoparticles while still in circulation [22,23,24]. Understanding the physiological interactions of circulating immune cells with metallic nanoparticles and determining where these nanoparticles ultimately reside in the body post intravenous administration will be critical for further clinical translation. Quantitative, label-free, real-time multiphoton intravital microscopy (MPIVM) procedures will be a valuable tool moving forward if the fundamental biological processes related to nanoparticle circulation half-life and extravasation are to be uncovered. Critically, label-free techniques circumvent the need for exogenous labels.

Nanoparticle visualization and tracking in vivo has always been challenging as a result of their submicroscopic size. The new luminescence imaging approach we utilize has several advantages over current nanoparticle measurement standards. Although NP circulation half-lives have been previously reported by taking subsequent blood draws followed by induction coupled plasma mass spectrometry (ICP-MS) analysis, this technique can be challenging, and important time points can be missed [25]. Subsequent blood draws can be difficult in mice, as the drawing volumes and frequency allowed are limited by strict Institutional Animal Care and Use Committee (IACUC) guidelines. The blood draw itself can also be challenging, as it may take several seconds to minutes to draw the appropriate volume needed for nanoparticle (NP) detection. By directly visualizing nanoparticles in circulation, multiphoton label-free imaging has the potential to avoid these pitfalls and provide a more accurate representation of minute changes in the blood concentration of nanoparticles.

Here, we report on a label-free gold nanoparticle imaging method that utilizes a multiphoton intravital microscopy (MPIVM) technique. This technique is capable of detecting single gold nanoparticle using multiphoton excitation, which induces broad-band upconverted luminescence, circumventing the need for exogenous labeling [26,27,28,29,30]. Luminescent properties associated with metals have yet to be applied successfully in vivo to track nanoparticles, flowing within the vasculature in real time [29,30]. We highlight a novel method utilizing the multiphoton luminescent properties of gold to visualize and quantify the particles within the vasculature. This label free imaging technique specifically avoids the perturbation of biological systems with exogenous fluorophores and instead utilizes the intrinsic contrast of the vascular environment and the gold nanoparticles themselves. This technique uses dual-laser multiphoton stimulation of gold nanoparticles and surrounding vasculature to produce images with label-free contrast from both vasculature and nanoparticles. In addition, the high frame rates used offer a unique opportunity to obtain blood concentration curves of gold nanoparticles non-invasively. This work offers the nano-community a new imaging approach for visualizing and tracking label-free gold nanoparticles in vivo.

## 2. Materials and Methods

### 2.1. Nanoparticle Fabrication and Characterization

Gold–silica nanoparticles were provided by Cabot Corporation (Boston, MA, USA); each particle comprised of a gold core of approximately 60 nm coated with a thiloated silica layer of approximately 35 nm, resulting in a total particle diameter of ≈130 nm (Supplementary Figure S1). Particles were suspended in Milli-Q water and diluted to the desired injection concentration. Raw gold nanoparticles were fabricated in house via the Turkevich method for gold nanoparticle synthesis [31]. Raw silica nanoparticles were also synthesized in house via the Stöber process [32]. The final concentration and size of all nanoparticles were quantified via nanoparticle tracking analysis on a Nanosight NS300 (Malvern Instruments, Malvern, United Kingdom) and verified via UV-vis spectroscopy on a Cary 60 UV-Vis spectrometer (Agilent Technologies Santa Clara, CA, USA) and electron microscopy imaging. Toxicity studies have been performed by our group and been previously published [33].

### 2.2. Animal Experiments and Injections

Female 8-week-old NU/NU (Crl:NU-Foxn1^nu^) nude mice (Charles River Laboratories, Wilmington, MA, USA) were used for all imaging studies. Intravenous injections of gold nanoparticles consisted of particles in 100 µL volume. To evaluate particle concentration and pharmacokinetics in vivo, mice were injected with a bolus dose via tail vain injection through a catheter. Following the injection, catheter lines were flushed with additional PBS in order to ensure the full dose was administered. All procedures performed on the animals were approved by the University’s Institutional Animal Care and Use Committee (IACUC #20847) and were within the guidelines of humane care of laboratory animals. 

### 2.3. Scanning Electron Microscopy Images of Gold Nanoparticles

Nanoparticles were drop cast onto etched silicon finder grids (Ted Pella Inc., Redding, CA, USA) in order to provide a conductive substrate with surface features visible to both multiphoton and scanning electron microscopes. Then, samples were imaged using a Nova NanoSEM 450 Field Emission Scanning Electron Microscope (Field Electron and Ion Company, Hillsboro, OR, USA) at 10 kV with a spot size of 4 in the immersion detector mode. Images were taken at low (≈2500×) and high (−15,000×) with fields of view ≈ 80 μm and ≈14 μm square, respectively. These magnifications were chosen to maximize the number of possible nanoparticles to later visualize using multiphoton microscopy. Nanoparticle shells were readily identifiable in the field by their core metallic structure. Corners of the etched grid were primarily imaged to be used as fiduciary features for co-registration between scanning electron and multiphoton images.

### 2.4. Preparation and Multiphoton Microscopy of Nanoparticle Samples

Silicon grids with drop-cast gold nanoparticles were mounted using Cytoseal 60 (Richard Allan Scientific Co., San Diego, CA, USA) and coverslipped. Images and emission spectra acquisition of gold nanoparticles on silicon finder grids was performed using a Leica TCS SP8 multiphoton microscope (Leica Microsystems, Wetzlar, Germany). Grid regions previously identified via SEM were imaged using a HC PL APO 63×/1.40 OIL CS2 objective (Leica Microsystems, Wetzlar, Germany). Tiling was used to image larger grid areas; then, the scanning region was reduced to gather the emission spectrum and to gather images for co-registration. Spectral data from gold–silica nanoparticles were acquired with 10 nm spectral resolution. A bleaching study was performed by drying a solution of gold–silica nanoparticles and fluorescein onto quartz slides and illuminating the field of view and then shifting the area of illumination. Gold and fluorescein intensities were recorded via specific region of interest (ROI) selection.

### 2.5. Scanning Electron/Multiphoton Microscopy Image Correlation

Image registration was performed manually by using the large etched corner features of the silicon finder grids. Images were first rotated and aligned using the corners; then, fine adjustment was performed by registering multiple nanoparticles or clusters of nanoparticles in the field of view. Identification of three or more individual points in combination with grid corners reliably co-registered SEM and multiphoton (MP) images.

### 2.6. Optical Setup for In Vivo Multiphoton Microscopy

The in vivo multiphoton imaging in this study was performed on a Nikon A1R HD upright multiphoton confocal microscope (Nikon Instruments, Inc., Melville, NY, USA). Two laser lines were utilized to maximize the signal generated from tissue in addition to nanoparticle luminescence. The laser system was provided by Coherent (Coherent, Palo Alto, CA, USA) and the source had a repetition rate of 80 MHz with a tunable wavelength from 800 to 1300 nm. Vasculature was illuminated at 920 nm and 1040 nm to maximize tissue autofluorescence and second harmonic generation along with gold nanoparticle emission. A CFI75 25X 1.1NA water immersion objective was used for imaging (Nikon Instruments, Inc., Melville, NY, USA). Laser power was 1.3 mW at the source, with a 30–35% loss through the system. Three-channel acquisition was used to maximize the signal collection from the broadband upconverted luminescence from nanoparticles. Initial studies indicated that frame rates above 200 frames per second were necessary to collect individual particles without blurring. The 1K resonant scanner on the A1R system was used to acquire the high frame rates required for video analysis of particle flow with a reduced scan area to increase the frame rate.

### 2.7. In Vivo Imaging

Mice were anesthetized with 2–3% isoflurane delivered at 100% oxygen at 1–2 L/min through a vaporizer. Once anesthetized, catheter lines were inserted into the tail vein, and the animals were positioned on the upright imaging table. Mice ears were temporarily attached to an imaging plate with double-sided tape to minimize movement during the imaging sessions. GenTeal (Alcon, Geneva, Switzerland) was used for immersion lens imaging in order to match the refractive index of water but with a solution that was viscous enough to remain in place during the imaging session. Once secured, imaging began prior to injection to locate a suitable vessel with flow rates between 100 and 500 μm/s. This flow rate allowed for gold nanoparticles to be easily tracked during post processing. Imaging channels were chosen to maximize the visibility of gold nanoparticle luminescent emission in vivo and second harmonic generation from the vasculature. Fifty nm bandpass filters at center wavelengths 446, 525, and 575 nm were selected for in vivo imaging. Mice were repositioned as necessary to align vessels in the orientation of the optimal imaging frame. Once a suitable vessel was located and the animal was positioned to maximize the length of the vessel in frame, video acquisition commenced followed by nanoparticle injection. Video was acquired for 30 min following injection. Vessel sizes were noted and flow rates were calculated in later stages of image processing. Scaling was necessary to accurately reconstruct the concentration curves when comparing separate mice and vessels. Without flow scaling, peak concentration is artificially inflated in a larger, faster flowing blood vessel due to a larger blood sample volume being analyzed over time.

### 2.8. Image and Video Processing

Nikon Instrument Software (NIS) Elements AR Version 4.30.01 (Nikon Instruments, Inc., Melville, NY, USA) was used for the initial video and image processing. Videos acquired from the particle injection studies were cropped to the vessel edges in order to efficiently proceed with particle tracking. Channel intensities were calibrated in this instance to maximize the visible signal from gold nanoparticles and minimize vasculature background. An elements de-noising process was applied to all video frames to reduce vessel intensity from red blood cells; then, a binary threshold was defined using a combination of channel intensities to set the threshold. This was necessary for reducing tracking errors, since the particles’ luminescence was referenced across channels to confirm the presence of a particle when applying the binary threshold. Particle luminescence was sufficiently bright in all vessels to accurately binarize the vessel flow for the nanoparticles. Particle tracking was accomplished within NIS elements via tracking of the binarized particles in the vessel flow video. Tracking parameters were adjusted to remove false positives and negatives using particle speed and size as criteria in determining a particle track. 

### 2.9. Video Processing and Particle Quantification (Statistics)

Raw tracking data were exported from NIS Elements into a custom Python-based Jupyter Notebook for data processing (Python.org, Python Version 3.3). Particle flow counts were binned to 1-s intervals and then standardized with a rolling average for visual analysis. All other data processing was performed in Microsoft Excel 2016 (Microsoft, Redmond, WA, USA). Still images in the text were extracted from video frames taken during imaging sessions and enhanced for contrast and clarity.

## 3. Results

### 3.1. Multiphoton Luminescence Properties of Gold Nanoparticles

**Figure 1:**
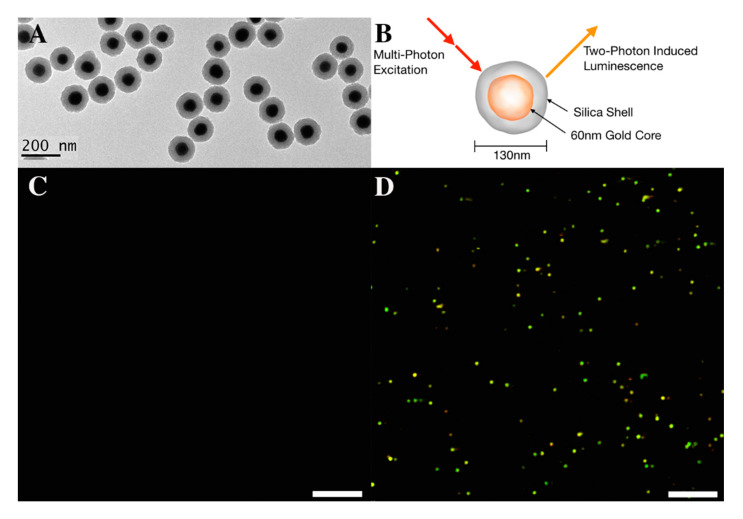
Label-free characterization of multiphoton-induced luminescence from gold–silica nanoparticles. (**A**) TEM image of 130 nm gold–silica nanoparticles. The silica shell is seen as the lighter gray band around a darker 60 nm gold core. (**B**) Diagram of proposed multiphoton luminescent effect in the gold–silica nanoparticles. Note that luminescence is generated only from the gold core. Images in (**C**,**D**) were acquired with gold–silica NPs prepared on a glass slide. Individual particles are highlighted in a golden hue due to the composite image taken from acquisition channels 446, 525, and 575 nm. Scale bars in panels C and D represent 10 μm.

Gold luminescent production was first observed in gold–silica nanoparticles containing a Raman active layer previously used for surface-enhanced Raman spectroscopy (SERS) imaging by our group (Figure 1) [6,34]. In order to confirm that the luminescence observed was a function of the gold cores as opposed to the gold–silica interface or Raman active component in our nanoparticles, we conducted the following experiments. We evaluated each of the following on slides: (1) 60 nm gold nanoparticles, (2) 130 nm pure silica nanoparticles, and (3) 126 nm gold-silica Raman active nanoparticles (consisting of ≈60 nm gold core and ≈35 nm silica shell) (Figure 1A).

Particles were aliquoted onto quartz slides and dried in place to minimize convection and particle movement due to heating when under laser illumination. Laser wavelengths of 920 nm and 1040 nm were chosen with the aim of maximizing both nanoparticle luminescence (920 nm) and second harmonic generation (SHG) from vasculature (1040 nm) with pulse width varying between 100 and 110 femtoseconds dependent on wavelength. Figure 1 depicts images of the gold–silica nanoparticles when under single photon (Figure 1C) and multiphoton (Figure 1D) illumination conditions. Notice how the gold silica nanoparticles are only detected under the multiphoton condition supporting a luminescence effect generated by multiphoton illumination.

To verify that the silica shell of the nanoparticles did not influence the luminescent production of the gold, raw silica nanoparticles were also excited under linear single-photon and non-linear multiphoton conditions. Subsequently, the silica particles did not produce detectable luminescence under either condition. Raw gold nanoparticles particles (60 nm citrate stabilized without a silica shell) were also evaluated and did produce luminescence under the same multiphoton conditions, confirming that luminescent production was a function of the gold nanoparticles and not the silica shell (Supplemental Figure S2).

**Figure 2:**
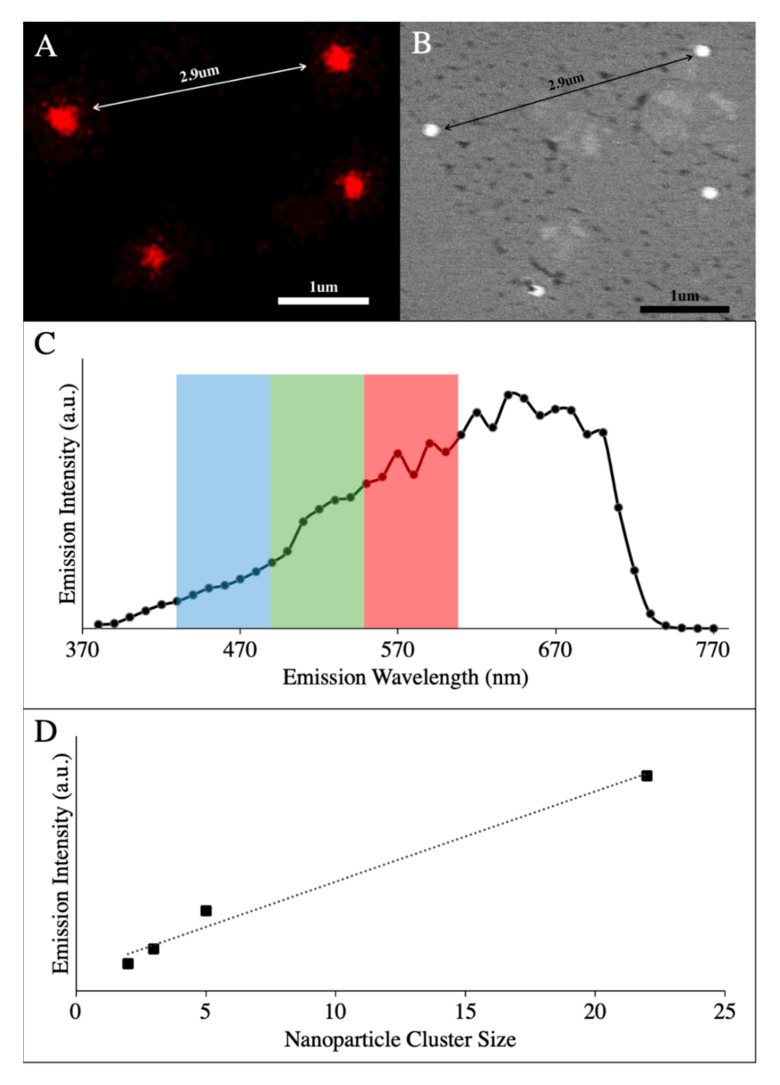
Correlative SEM and MP (multiphoton) images of gold nanoparticles (**A**) Multiphoton emission from gold–silica nanoparticles at 600 nm. (**B**) SEM image of the same four gold–silica nanoparticles taken at 15,000× magnification. The dense gold cores are present in white. (**C**) Emission spectrum of gold nanoparticles with overlaid imaging channels from in vivo imaging. (**D**) Average emission intensity from small clusters of gold–silica nanoparticle clusters for 600–700 nm. Note the linear increase in emission over a wide variety of clusters sizes. Extinction spectra for gold–silica nanoparticles can be found in Supplemental Figure S4. Emission data for the clusters can be found in Supplemental Figure S5.

To demonstrate single nanoparticle luminescence detection, gold–silica nanoparticles were drop cast onto silicon finder grids and examined under SEM. Nanoparticles were identified with SEM (Figure 2A), and the same sample was then imaged by multiphoton microscopy at 1000 nm excitation where single gold nanoparticles produced enough luminescence for identification and co-registration (Figure 2B). Note that the gold–silica nanoparticles produce a broad-spectrum luminescence (Figure 2C), which is registered across three acquisition channels with center wavelengths of 425, 525, and 575 nm. This results in the “golden” hue as seen in Figure 1D when the channels are merged into a composite image. Emission characterization was also performed and at 1040 nm, multiphoton luminesce is driven by a 4-photon process (Supplementary Figure S3). Although this imaging technique can detect single nanoparticles, as demonstrated in Figure 2A, single spots identified as nanoparticles when viewed on a multiphoton microscope are inherently diffraction limited. Therefore, a cluster of particles will have approximately the same diameter as a single nanoparticle until much larger aggregates are viewed. However, we observed a linear trend where emission intensity linearly increases (Figure 2D) with respect to the number of gold–silica nanoparticles in the cluster, and we did not observe appreciable emission changes when clusters were imaged (Supplemental Figure S5). This linear trend has the potential to enable the extraction of quantitative information about the nanoparticles we track, and it is further being investigated in our lab. Finally, this broadband luminescence has also been observed at lower excitation wavelengths and is consistent with the multiphoton luminescent properties previously described [30,35].

A photobleaching study was also performed to compare the luminescent properties of the gold–silica nanoparticles to the fluorescence properties of the traditional clinical fluorophore, fluorescein. Gold nanoparticles and fluorescein dye were separately applied and dried onto quartz slides followed by illumination at approximately 400 mW and 278 mW, respectively. ROIs from the illuminated area were analyzed for intensity loss over time that would indicate photobleaching. Gold nanoparticle luminescent production did not decrease over time when using a 920 nm laser excitation at 400 mW laser power over the course of 60 s (Supplemental Figure S6). The fluorescein sample did undergo a significant loss of signal intensity over the course of the exposure at 278 mW, and acquisition was stopped at 60 s. Since this luminescence effect is intrinsic and did not undergo photobleaching, extended imaging sessions (>20 min) were then performed to evaluate the gold nanoparticle’s true circulation half-life. 

### 3.2. Visualization of Gold Nanoparticles In Vivo

**Figure 3:**
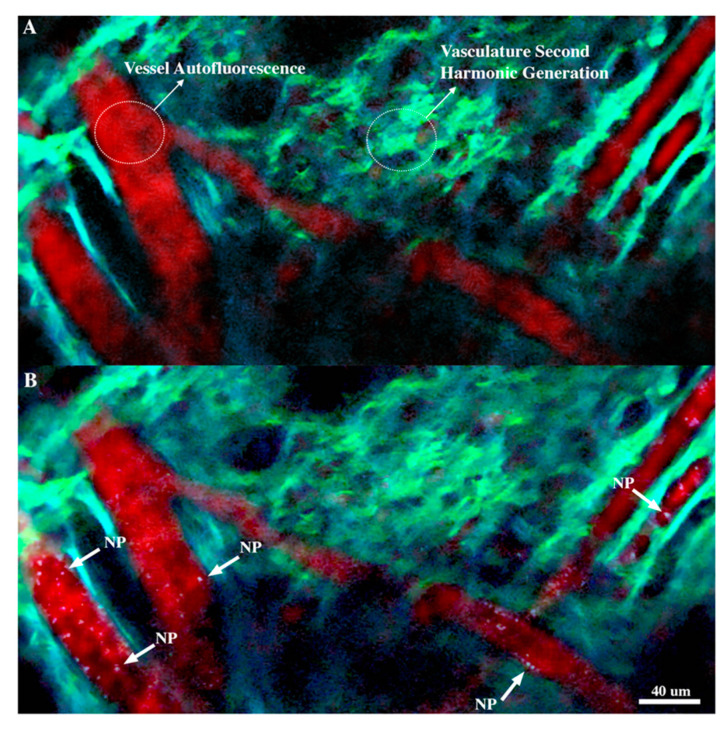
Label-free wide field views from video acquisition of gold–silica nanoparticle flow in ear vasculature. (**A**) Vascular network image prior to gold–silica nanoparticle injection. Background vessel autofluorescence is seen in red, while tissue autofluorescence and SHG (second harmonic generation) are observed in cyan. Red blood cell shadows can be observed as dark spots within the vessels. Imaging channels are a composite of three channels from 450 to 600 nm. (**B**) Vascular network image following gold–silica nanoparticle intravenous injection. Gold–silica nanoparticles appear as white spots within the vessels, and selections are highlighted by white arrows. These nanoparticles are completely label-free and are only producing a signal from the multiphoton luminescent process. Both still images portrayed here are taken from the video acquisition available in Supplemental Video 1.

With the initial gold luminescent parameters acquired, we investigated a method for imaging in a living animal model so that particles could be tracked and quantified. This began with the mouse ear adhered to an imaging plate (Supplemental Figure S7) and macro-scale vascular networks imaged to locate suitable vessels for nanoparticle visualization (Figure 3A). Vessels with linear paths through the frame and minimal branching locations were selected to minimize error in nanoparticle blood counts. Additionally, vessels were oriented perpendicular to the resonant scanning vertical refresh direction. This was to ensure that particles would not be illuminated twice in any one video frame as the vertical raster is considerably slower than the horizontal raster speed. Figure 3 shows a sample wide field view (460 μm × 230 μm) with a vascular network and gold–silica nanoparticles highlighted by white arrows (Figure 3B). Strong second harmonic generation and autofluorescence was seen in the ear vasculature, which is represented in cyan and red, respectively. It is important to note these images are completely label free, with all contrast being produced from either the gold nanoparticles or the vascular environment itself through SHG and autofluorescence. A video of this figure showing nanoparticles arriving in real time can be found in Supplemental Video 1.

**Figure 4:**
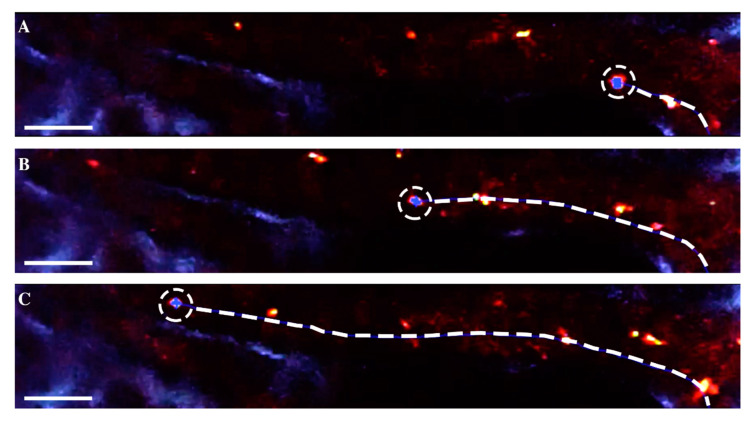
Sample particle tracking of an individual gold–silica nanoparticle within vasculature. (**A**–**C**) Nanoparticles are identified through the co-registration of luminescence in three imaging channels: 446, 525, and 575 nm. The nanoparticle is position tracked through the various frames of the video acquisition in white dashed circles, and blood velocity is recorded simultaneously. Particle tracking software assigns blue dots overlaid on the tracked particle, creating the binary layer formed from the three imaging channels. Refer to Supplemental Video 2 for a video acquisition of nanoparticle tracking. Scale bar represent 40 μm.

Preliminary in vivo tests demonstrated that luminescent production from the nanoparticles was sufficient at low enough laser power on the tissue to perform a quantitative analysis of particles within the blood stream with a reduced field of view and higher frame rates (Figure 4). Laser power for the 920 and 1040 nm channels were approximately 110 and 62 mW, respectively. Figure 4A–C highlights the reduced field of view necessary to increase frame rates for accurate particle visualization and tracking along with the linear vessel structure. Orientation of the raster scan head on the imaging system was critical as the frame refresh direction had to be aligned perpendicular to the direction of blood flow. This orientation minimized the “doubling” or blurring of particles, which occurs when the particle is registered twice as the frame is collected at lower frame rates (Supplemental Figure S8). Frame rates during particle flow-in were set at 226 frames per second (fps), which balanced the field of view and frame rate adequately for the particle tracking imaging session. This frame rate was experimentally determined to allow for the best imaging quality while also allowing for quantification of the particles flowing. Frame doubling occurs at lower frame rates due to the laser raster scan illuminating a gold nanoparticle twice in the acquired video frame. This imaging artifact precludes accurate nanoparticle tracking in free-flowing vasculature, especially when multiple nanoparticles are present in the frame.

The autofluorescence and second harmonic generation was used to identify vascular regions with a reasonable flow rate and path. Dual excitation wavelengths of 920 and 1040 nm were selected to optimize gold luminescence production (920 nm) and SHG and autofluorescence from the vasculature (1040 nm). Collagen is presented in green from the 525 nm green channel, while autofluorescence from the vasculature walls is presented in the red channel (575 nm) (Figure 3A). Although the gold particles produced a much greater signal as compared to the vasculature, the SHG from the tissue provided more than enough contrast to highlight vasculature features and locate vessels of interest.

### 3.3. Particle Tracking to Asses Vascular Kinetics

Optical tracking of nanoparticles to determine blood concentration is a highly desirable technique especially compared to methods such as periodic blood draws. Time-lapse MPIVM offers systemic analysis and real counts of individual particles as they flow in the bloodstream. With the imaging parameters taken from the initial imaging sessions, we assessed the viability of obtaining in vivo kinetic vascular data using the imaging technique. Mice were arranged in an identical configuration with ears adhered onto an imaging stage (Supplemental Figure S7). Two gold–silica nanoparticle injections were prepared (Figure 4B) with concentrations of 2 and 9 nM. The concentration ratio of these two injections was also quantified for later comparison with measured time points within the vasculature. Then, individual mice were injected with either of the two known concentrations of nanoparticles (2 or 9 nM).

**Figure 5:**
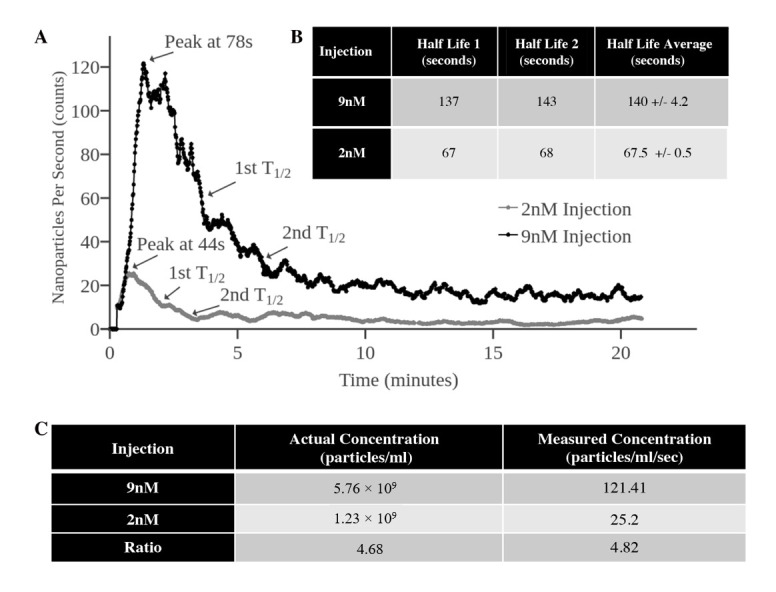
Blood concentration data derived from collective nanoparticle tracking within vasculature. (**A**) Nanoparticle blood concentration curves derived from two different nanoparticle injections, normalized to blood volume and velocity within individual vessels. Peak flow concentration is noted in both curves along with the concentration half-lives. (**B**) Table of nanoparticle blood half-lives for each injection. Note that the first and second half-lives remain consistent for both injections. (**C**) Ratiometric comparison of particle counts observed at peak concentration within the animal model and the known pre-injection concentration. The pre-injection concentration ratio and the observed in vivo concentration ratio between the two injections remain relatively consistent.

Flow data were compared first by performing a ratiometric analysis of pre-injection concentration and then correlated with post-injection peak counts per second through the vessel. Figure 5 shows the scaled particle flow count data over time for both the 2 and 9 nM injections. The time at peak concentration in vivo was determined by the peak counts per second flowing through the vessel in all trial runs and scaled by vessel volume and blood velocity. This scaling gave a more accurate representation of the blood concentration, as blood flow differences inherently change the number of particles seen in a given period of time. Blood half-lives were calculated starting from the peak nanoparticle concentration observed and reported as the time taken for the average blood flow counts to reach half the original value. Note that the blood half-lives in Figure 5B do not remain the same when the injection concentration is increased. This may be due to a reticuloendothelial system (RES) blockade effect that has previously been reported with the high dose administration [25]. The ratio of particle concentration in the injected dose and the observed concentration of the flowing nanoparticles is nearly equivalent, as seen in Figure 5C. The correlation of these values in vivo was taken at the peak concentration observed in order to reduce the differences in uptake as the nanoparticles were eliminated from the bloodstream. This correlation suggests that our MPIVM imaging and processing technique would be well suited for further in vivo vascular kinetic analysis of gold nanoparticles.

## 4. Discussion

Here, we have demonstrated a viable quantitative label-free method for the in vivo tracking and analysis of gold nanoparticles via MPIVM. By utilizing the intrinsic luminescence of gold nanoparticles combined with second harmonic generation and autofluorescence of biological tissue, high-resolution imaging of these structures was feasible without exogenous labels. Since this luminescence effect for gold is intrinsic and does not undergo photobleaching, extended imaging sessions (>20 min) were possible to evaluate gold nanoparticles’ true circulation half-life. This MPIVM technique has significant potential to facilitate biodistribution, extravasation, and a tumor uptake studies of gold nanoparticles without labeling, leaving the biological system unperturbed. The further development and translation of nanoparticle-based imaging techniques into the clinic necessitate a better understanding of the pharmacokinetics and pharmacodynamics of metallic nanoparticles in vivo and our technique has potential to facilitate this work.

As with every imaging technique, there are both advantages to exploit and limitations to consider when designing an experiment and deciding which research questions to address. It is important to note that the imaging experiments performed were carefully designed to answer a fundamental question on the feasibility of the direct visualization of gold nanoparticles in circulation while working within the imaging capabilities of our proposed approach. One of the biggest advantages of utilizing this multiphoton luminescence imaging technique to track metallic nanoparticles is that it is entirely label-free and has high frame rate capabilities to achieve real-time imaging. Our results demonstrate that we can detect single gold nanoparticles with our multiphoton microscope setup in a controlled silicon grid environment. Figure 2 reveals an SEM image overlaid with the corresponding multiphoton image to indicate that a single gold nanoparticle can provide enough luminescence for our instrument to detect. The imaging technique is also highly specific due to the metallic nanoparticle’s unique spectral luminescence emission signature (Figure 2). A limitation of the proposed imaging technique is its spatial resolution of ≈450 nm. Although we have shown the ability to sensitively detect single nanoparticles, we are limited in resolving them from neighboring nanoparticles within ≈450 nm. However, given that we observed a linear increase in emission intensity when particles are clustered, the technique may still be viable for the quantitative analysis of label-free particles and should be further investigated.

Prior studies have reported on the multiphoton luminescent effect from gold nanoparticles and even utilized the effect to image gold nanoparticles in vitro [35,36,37]. However, cellular level imaging techniques only provide useful data on the uptake and cytotoxic effects of nanoparticles but lack many fundamental realities present during in vivo analysis. Although these studies have also alluded to the use of the luminescent effect in vivo, none have succeeded in creating quantitative in vivo data of free-flowing gold nanoparticles, which is likely due to the high frame rates and laser scan speed needed. Only recently have commercial systems become available with resonant scanners capable of frame rates necessary to record nanoparticle flow within the blood. To our knowledge, the frame rates in this study are the first to be utilized with a label-free non-linear multiphoton imaging technique for tracking gold nanoparticles in free-flowing blood. Although the visualization of gold nanoparticles at frame rates lower than 100 fps has been demonstrated previously, significant imaging artifacts including the “doubling” of particles in the frame are present. In our work, frame rates over 200 fps were required to visualize nanoparticles without these artifacts. Due to irregularities observed in blood flow rates when assessing multiple vessels, frame rates up to 226 fps were used in this study to ensure artifact-free images.

Utilizing MPIVM for nanoparticle pharmacokinetic analysis has distinct advantages over traditional blood draw methods or radiolabeling—specifically, that it is possible to track particles non-invasively in real time and in an unperturbed environment. MPVIM also allows for the multiplexing of fluorescent nanoparticles and luminescent nanoparticles with the simultaneous label-free visualization of tissues (Supplemental Video 3). The broadband luminescence of gold nanoparticles results in gold nanoparticles registration across multiple imaging channels, whereas fluorophores or the intrinsic autofluorescence of biological tissue primarily occur in one imaging channel. The spectral separation of these imaging agents permits the simultaneous assessment of far more biological entities than would be possible when using only fluorescent small molecules. This multiplexing ability opens an entirely new door toward understanding how metallic nanoparticles interact with different cell populations within the vasculature (i.e., circulating leukocytes) and may provide a better understanding of how and when they are cleared from the blood in real time.

Finally, it is important to note that this luminescence imaging technique is not limited to gold and holds promise for investigating other metallic nanoparticles such as titanium dioxide and silver in a label-free in vivo environment. Prior work by Dietzel et al. has demonstrated that these nanoparticles exhibit multiphoton luminescence in addition to second and third harmonic generation [26]. However, these luminescent properties have not been fully exploited in vivo and could provide ways to interrogate the long-term safety of these particles through direct visualization. Silver and titanium dioxide nanoparticles are some of the most widely used nanomaterials in industry ranging in applications from cosmetics to food additives and chemical catalysts. Titanium dioxide is of particular concern for consumer safety because of its use in sunscreen and subsequent long-term exposure with prolonged use. In addition, recent work on the synthesis and use of quantum clusters using gold and silver have produced promising results for fluorescent imaging [38]. These ultra-small nanoparticles, typically less than 2 nm, can produce tunable emission based on their size and could be excellent candidates for our imaging technique especially given their very low toxicity.

## 5. Conclusions

In summary, we have demonstrated the viability of multiphoton luminescence produced from gold nanoparticles for real-time imaging in vivo. The co-registration of SEM and multiphoton microscopy images revealed that emission from single gold nanoparticles was sufficient for the detection, localization, and spectral emission characterization. In vivo tracking of free-flowing particles with MPIVM allowed for label-free and non-invasive blood concentration analysis. Exploiting the ultra-high frame rates and label-free visualization characteristics of this imaging technique could unveil a wealth of real-time data about nanoparticle interactions that was not previously possible. Further investigation using this visualization technique could allow for an overall improved fundamental understanding of the biological interactions taking place in a living model with these metallic-based nanoparticles to promote their clinical translation.

## Supplementary Materials

The following are available online at https://www.mdpi.com/2079-4991/10/11/2239/s1, Figure S1. Gold–Silica Nanoparticle Size Distribution, Figure S2. Bare Gold Nanoparticle Multiphoton Emission Characterization, Figure S3. Gold Silica Nanoparticle Emission Characterization, Figure S4. Nanoparticle Extinction Spectra, Figure S5. Gold Silica Nanoparticle Cluster Emission Characterization, Figure S6. Gold Nanoparticle and Fluorescein Bleaching Study, Figure S7. Mouse Ear Imaging Setup, Figure S8. MPIVM Image Doubling Effects While Tracking, Video S1: Wide Field In Vivo Imaging of Gold Nanoparticles, Video S2. Gold–silica Nanoparticles in vivo Tracking Demonstration, Video S3: Multiplexing Capabilities of Fluorescent Imaging Agents and Gold Nanoparticles.
