# Supplementary material for: Label-Free Visualization and Tracking of Gold Nanoparticles in Vasculature Using Multiphoton Luminescence
Source: Nanomaterials (Basel). 2020 Nov 12;10(11):2239. doi: 10.3390/nano10112239 (PMC7696495; doi:10.3390/nano10112239)
Supplement: Supplementary file 1 [file nanomaterials-10-02239-s001.zip › nanomaterials-943294-2-SI.docx]

Supplementary Materials

Label-Free Visualization and Tracking of Gold Nanoparticles in Vasculature Using Multiphoton Luminescence

Sean Burkitt ^1^, Mana Mehraein ^2^, Ramunas K. Stanciauskas^3^, Jos Campbell^4^, Scott Fraser^5^, Cristina Zavaleta^6,^*

^1^ Department of Biomedical Engineering, University of Southern California, 1042 Downey Way, Los Angeles, CA 90089; Michelson Center for Convergent Biosciences, 1002 Child’s Way, Los Angeles, CA 90089; Bridge Institute, University of Southern California, 1002 Child’s Way, Los Angeles, CA 90089; sburkitt@usc.edu

^2^ Department of Biomedical Engineering, University of Southern California, 1042 Downey Way, Los Angeles, CA 90089; Michelson Center for Convergent Biosciences, 1002 Child’s Way, Los Angeles, CA 90089; mehraein@usc.edu

^3^ Nikon Instruments Inc., 1300 Walt Whitman Road, Melville NY 11747-3064; ramunas.stanciauskas@nikon.com

^4^ Department of Biomedical Engineering, University of Southern California, 1042 Downey Way, Los Angeles, CA 90089; Michelson Center for Convergent Biosciences, 1002 Child’s Way, Los Angeles, CA 90089; joscampb@usc.edu

^5^ Department of Biomedical Engineering, University of Southern California, 1042 Downey Way, Los Angeles, CA 90089; Michelson Center for Convergent Biosciences, 1002 Child’s Way, Los Angeles, CA 90089; Bridge Institute, University of Southern California, 1002 Child’s Way, Los Angeles, CA 90089; Department of Biological Sciences, University of Southern California, 3616 Trousdale Parkway, Los Angeles, CA 90089; sfraser@provost.usc.edu

^6^ Department of Biomedical Engineering, University of Southern California, 1042 Downey Way, Los Angeles, CA 90089; Michelson Center for Convergent Biosciences, 1002 Child’s Way, Los Angeles, CA 90089; Bridge Institute, University of Southern California, 1002 Child’s Way, Los Angeles, CA 90089; czavalet@usc.edu

***** Correspondence: czavalet@usc.edu


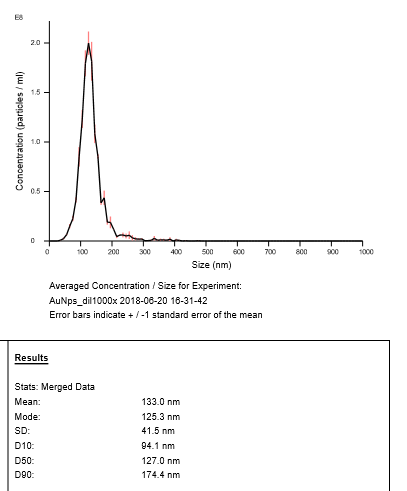


**Supplemental Figure S1.** **Gold-Silica Nanoparticle Size Distribution** Gold-silica nanoparticle size distribution determined by Nanosight NS300.


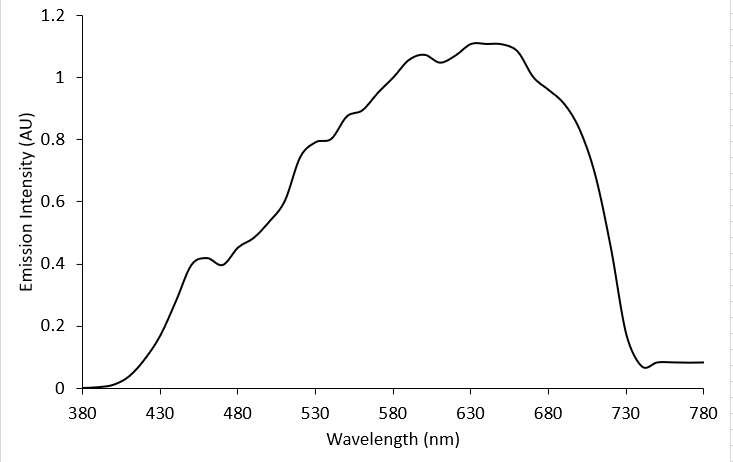


**Supplemental Figure S2.** **Bare Gold Nanoparticle Multiphoton Emission Characterization** 60nm gold nanoparticle emission at 1040nm excitation. Note that the emission spectrum is nearly identical to the gold-silica nanoparticle emission.


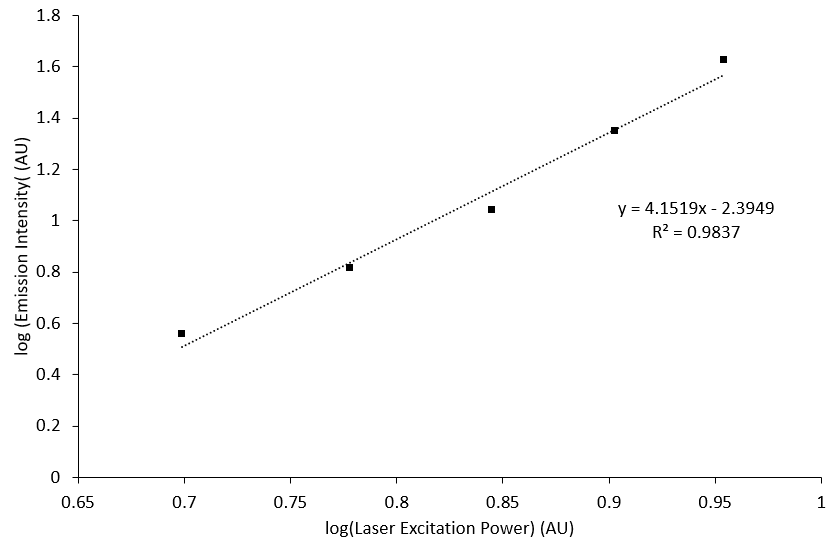


**Supplemental Figure S3.** **Gold Silica Nanoparticle Emission Characterization** Logarithmic plot of multiphoton induced luminesce of 130nm gold-silica nanoparticles (60nm gold core). The slope of the plot indicates a 4-photon process responsible for the light emission at 1040nm excitation. Emission was characterized at the peak 680nm emission.


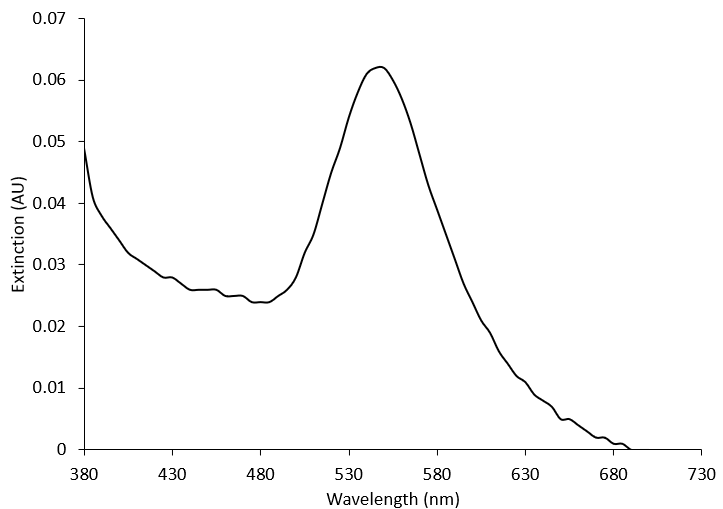


**Supplemental Figure S4.** **Nanoparticle Extinction Spectra** 130nm gold-silica nanoparticle extinction


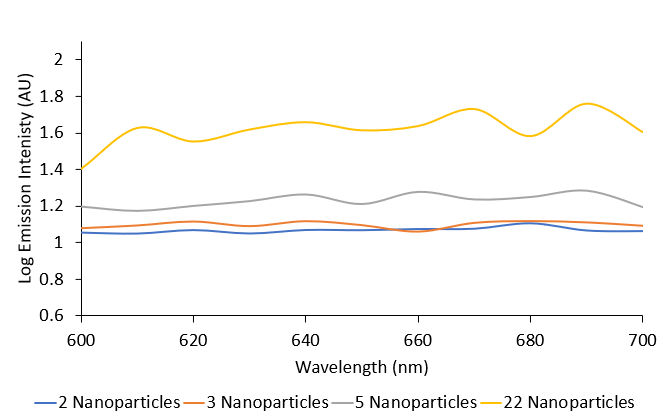


**Supplemental Figure S5.** **Gold Silica Nanoparticle Cluster Emission Characterization** 130nm gold-silica nanoparticle cluster emission from 600 to 700nm at 1040nm excitation. Emission for clusters of nanoaprticles minimally varies except for the linear intensit increase as the number of nanoaprticles in the cluster increases.


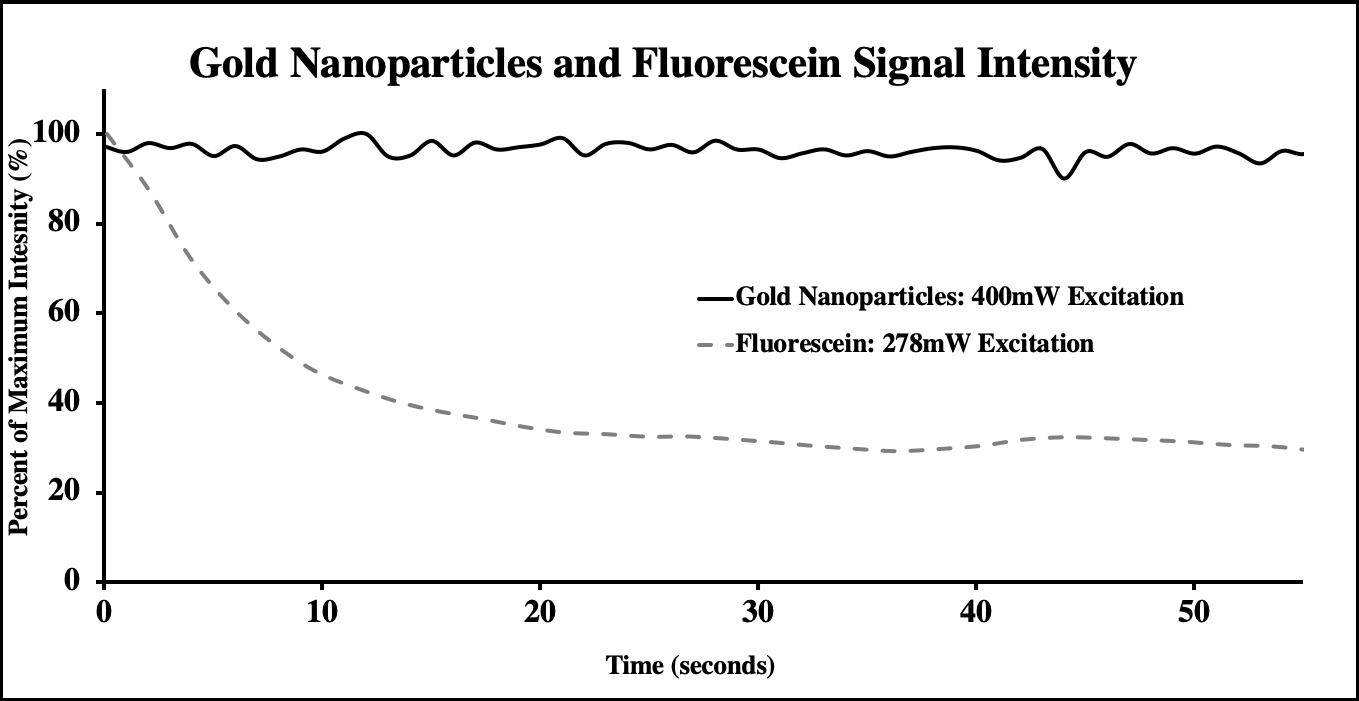


**Supplemental Figure S6.** **Gold Nanoparticle and Fluorescein Bleaching Study** Gold Nanoparticle and fluorescein bleaching study. Both gold-silica nanoparticle and fluorescein were dried on to quartz slides prior to illumination. Fluorescein was excited at 920nm and 278mW and bleaching was quantified via ROI within the frame. Gold Nanoparticles were illuminated at 400mW and 920nm and luminescent intensity quantified via ROI within the frame. Note that the gold-nanoparticle luminescent intensity does not decrease even after 60 seconds of continuous illumination at 400mW. This illumination time is significantly longer than what would occur in any in vivo imaging situation, especially within free-flowing vasculature.


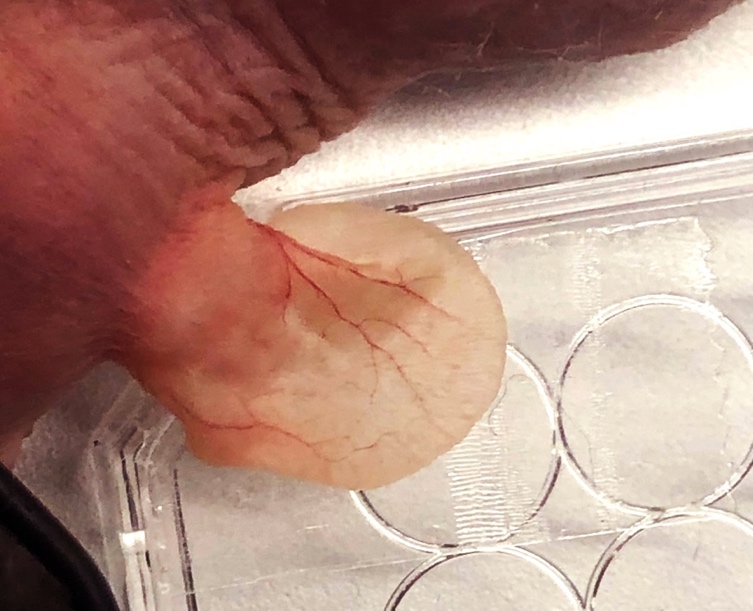


**Supplemental Figure S7.** **Mouse Ear Imaging Setup** Mouse ear imaging setup for visualization of gold-silica nanoparticles in vivo. Mouse ears were adhered to small shunt (96 well plate top) in order to provide better alignemnt with the upright microscope’s objective. Gentile was place on the ear for the immersion lens. Vsascualar networks in the ear were well suited for imaging due to the relatively clear tissue and thin tissue present, in addition to the high density vascualture.


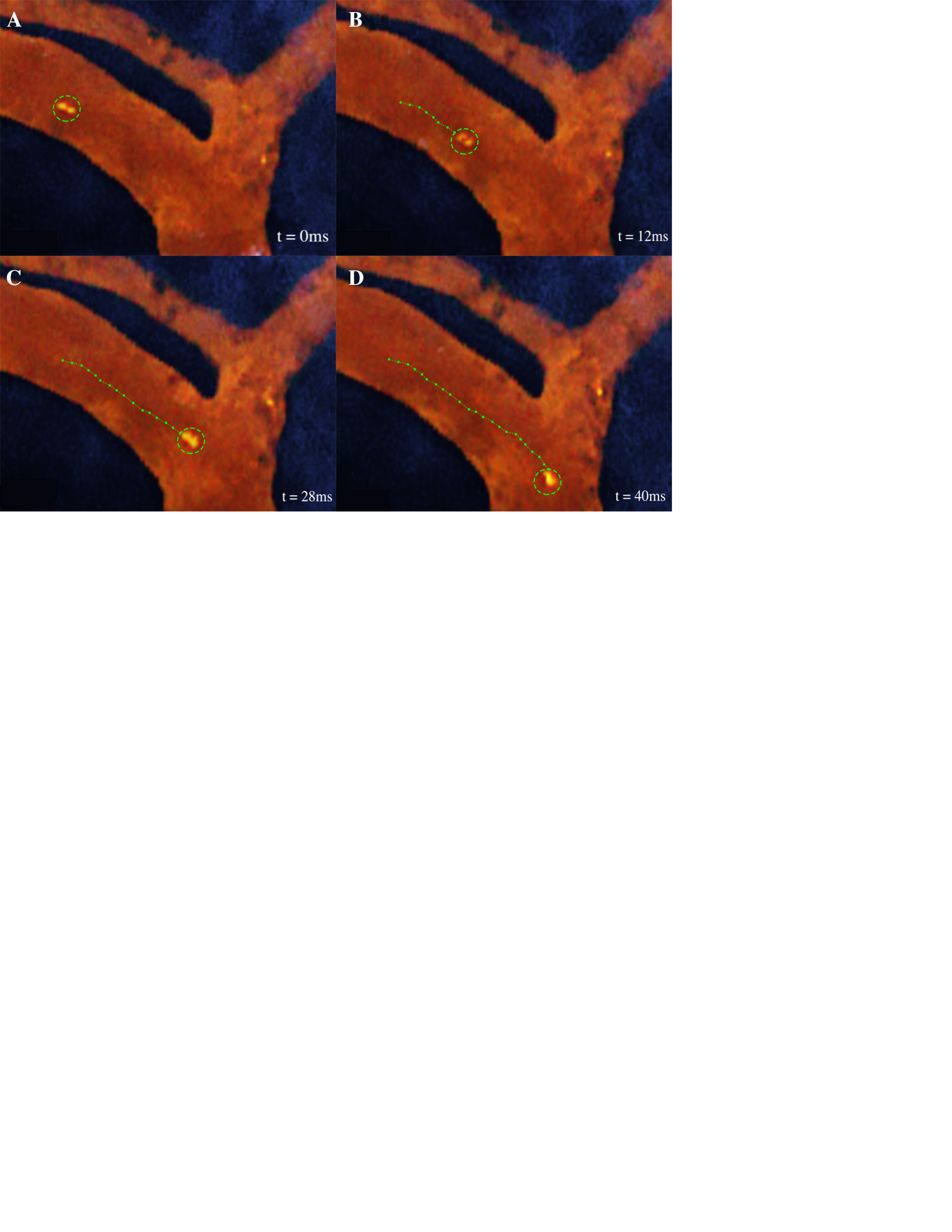


**Supplemental Figure S8.** **MPIVM Image Doubling Effects While Tracking** Particle doubling effect observed at reduced frame rates in vivo. A single gold-silica nanoparticle flowing through dyed vascualture (a-d). Doubling occurs within multiple frames due to both misalignment of the scan head relative to the blood flow direction in addition to the lower frame rates (60 fps). The doubling effect is reduced at this frame rate, but not fully eliminated until approximately 200 fps.

Supporting Video Descriptions:

**Supplemental Video 1**. **Wide Field *In Vivo* Imaging of Gold Nanoparticles** Gold-silica nanoparticle preliminary label-free in vivo visualization. Vascualr enviroment imaged prior to gold silica injection using SHG and autoflorescence of tissue for contrast primarily in the cyan and green imaging channels, 446nm and 525nm center wavelenght respectively. Gold nanoparticles are seen as white dots withing the vessels, progressing from the left side of the vascular enviroment to the right. Note that no additional fluorophores or imaging agents are present in the video. This larger field of view is approxiamtely 460 um x 230 um at a frame rate of 60 fps.

**Supplemental** **Video 2.** **Gold-silica Nanoparticles *in vivo* Tracking Demonstration**. Gold-silica nanoparticles are seen in free flowing vasculature with a single particle tracked (highlighted in blue) through 3 channel merged binarization. Speed of the particle is also shown below the vessel flow. Video was acquired at 226 fps.

**Supplemental** **Video 3**. **Multiplexing Capablities of Fluorescent Imaging Agents and Gold Nanoparticles**. Gold-silica nanoparticles, green fluorescent liposomes and a vaacular flourescent agent were injected into the animal to test spectral sepereation and the ability to distinguish multiple flourophores in vivo. As in prior label free video acquistions, there is strong SHG and autflourescne of the vascualture. Labeling of the vascualture was not performed with exogenous dyes. Gold-silica nanoaprticles are presented as purple spots in the vessel, green liposomes in green, in addidtion to the small molecule vacular dye which was injected and serves as background contrast in the vesseles themselves. Shaking within the frame is due to animal repsiration during the video acquistion. Video was acquired at 60 fps.
